# Ubiquitination of HLA-DO by MARCH family E3 ligases

**DOI:** 10.1002/eji.201243043

**Published:** 2013-02-11

**Authors:** Martin Jahnke, John Trowsdale, Adrian P Kelly

**Affiliations:** Division of Immunology, Department of Pathology, University of CambridgeCambridge, UK

**Keywords:** HLA-DO, MHC antigen presentation, Ubiquitination

## Abstract

HLA-DO (DO) is a nonclassical MHC class II (MHCII) molecule that negatively regulates the ability of HLA-DM to catalyse the removal of invariant chain-derived CLIP peptides from classical MHCII molecules. Here, we show that DO is posttranslationally modified by ubiquitination. The location of the modified lysine residue is shared with all classical MHCII beta chains, suggesting a conserved function. Three membrane-associated RING-CH (MARCH1, 8 and 9) family E3 ligases that polyubiquitinate MHCII induce similar profiles of polyubiquitination on DOβ. All three MARCH proteins also influenced trafficking of DO indirectly by a mechanism that required the DOβ encoded di-leucine and tyrosine-based endocytosis motifs. This may be the result of MARCH-induced ubiquitination of components of the endocytic machinery. MARCH9 was by far the most efficient at inducing intracellular redistribution of DO but did not target molecules for lysosomal degradation. The specificity of MARCH9 for HLA-DQ and HLA-DO suggests a need for common regulation of these two MHC-encoded molecules.

## Introduction

HLA-DO is a nonclassical MHC molecule that is closely related in sequence and structure to the HLA class II molecules DP, DQ and DR. It shares a similar degree of homology with these classical molecules as they do with each other, suggesting a common ancestry 1. It also shares conserved promoter elements with MHCII and requires CIITA, the master regulator of class II gene expression, for transcription 2. However, tissue expression of DO is much more restricted than for MHCII, with DO protein mainly detected in B cells but also reported in thymic epithelium and some DC subsets 3–5. HLA-DO is composed of alpha and beta chains that associate directly with HLA-DM early in biosynthesis. This pairing is essential for DO maturation 3, 6. In the absence of DM, DO dimers fail to assemble properly and locate predominantly to the ER. Consequently, DO is always expressed together with HLA-DM and the two molecules co-localise in late endocytic compartments 5, 7. Experimentally, the dependency of DO for DM can be circumvented by a proline to valine substitution in the membrane distal domain of DOα and this mutation provides a useful tool for the study of DO in the absence of DM 6.

Although the genes encoding DO were discovered soon after those for MHCII, its role in class II antigen presentation is not well defined. HLA-DP, -DQ and -DR function to present peptides to CD4-restricted T cells. They assemble in the ER with the chaperone molecule invariant chain (Ii) and traffic to endosomal compartments where Ii is degraded 8. A small fragment of Ii, CLIP, remains in the peptide-binding groove and is removed through interaction with the nonclassical molecule HLA-DM 9. The exact role of DO in MHCII antigen presentation remains uncertain but it does not associate directly with peptide. Instead it is proposed to function as a negative regulator of HLA-DM 10.

Direct examination of the interaction of DM and MHCII through crystallography has proven difficult. However, recent co-crystallisation of a DO/DM complex has shed light on the function of both DM and DO 11. These studies demonstrate that DO adopts an MHCII-like structure and associates in a side-by-side arrangement with DM. DO binds to the same sites on DM as those predicted to be involved in DM/DR interactions 11. Mechanistically, DM assists peptide release by disrupting key MHC-peptide interactions and it also stabilises MHCII in a peptide-receptive conformation 12, 13. In contrast, HLA-DO acts as a competitive inhibitor of DM and may allow “fine-tuning” of peptide loading during the process of affinity maturation 11, 14. This may be achieved by pH-dependent dissociation of DM/DO complexes thereby allowing “focusing” of peptide loading to late endocytic compartments 3. DO has also been shown to mediate redistribution of DM and MHCII to the limiting membrane of multivesicular bodies (MVB) 15, to limit DM sorting into exosomes 16, and to control B cells entry into germinal centers (GC) 17. However, there is still considerable uncertainty regarding its biological role.

Posttranslational modification of MHCII is required to efficiently control antigen presentation. Ubiquitination plays a key role in this regard by regulating the level of peptide-loaded MHC class II (pMHCII) complexes on the surface of professional antigen-presenting cells 18–20. In immature DCs, MHCII is sequestered in endosomes due to ubiquitination by membrane-associated RING-CH (MARCH1) 20. Upon activation by TLR stimuli, ubiquitination ceases, MARCH1 mRNA levels decrease, and pMHCII is redistributed to the plasma membrane 20. This allows cells to present on their surface a “snap-shot” of antigens present at the time of DC activation. Ubiquitination has numerous consequences for MHCII, including regulation of intracellular localisation and protein turnover. Given their close amino acid homology and overall structural similarity, we investigated if, like classical ­MHCII, HLA-DO was subject to posttranslational modification. Here we show that MARCH E3 ligases regulate DO directly through ubiquitination of a lysine residue present in the cytoplasmic tail of DOβ. They also influence trafficking of DO indirectly, probably through ubiquitination of components of the endocytic machinery.

## Results

### Residue K225 in the cytoplasmic tail of DOβ controls MARCH-induced internalisation of CD8-DOβ

**Figure 1:**
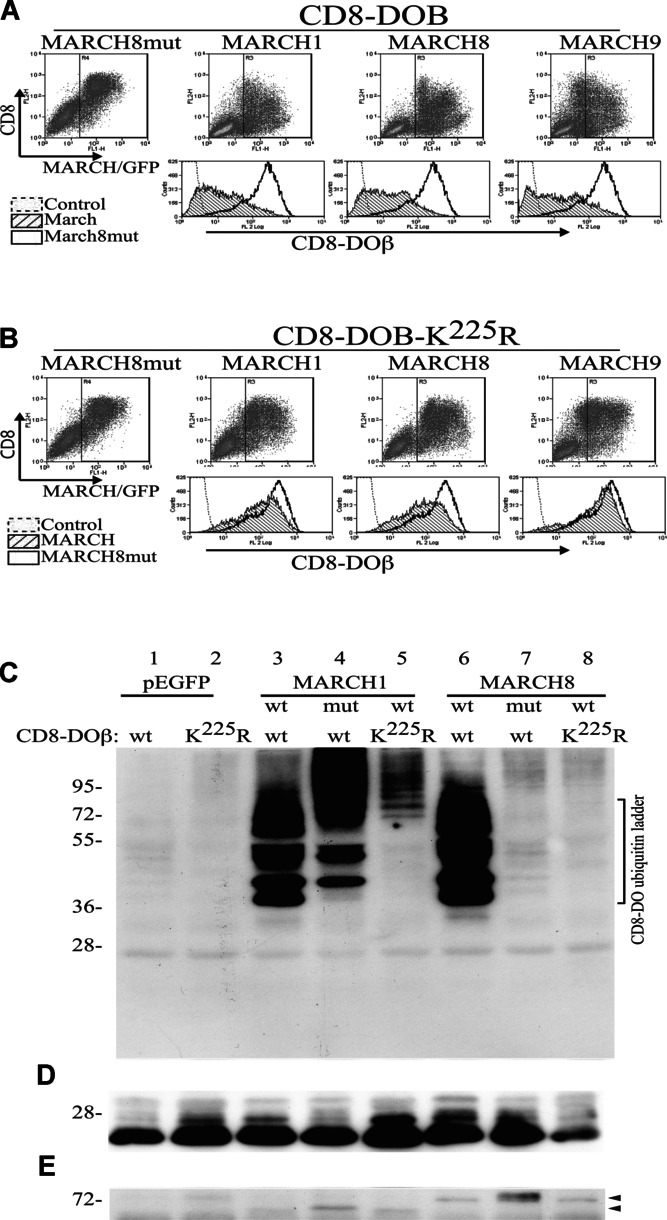
Surface expression and ubiquination of CD8-DOβ reporter molecules in the presence of MARCH family E3 ligases. HEK 293T cells were transfected with CD8-DOβ reporter constructs together with active and inactive MARCH-EGFP E3 ligase constructs. Cells were prepared for flow cytometry analysis and surface levels of CD8 determined with OKT8-PE (FL2). (A) Dot plots and histogram profiles showing wild-type CD8-DOβ surface expression in the presence of MARCH1, MARCH8, MARCH9 and catalytically inactive MARCH8. (B) As above but with CD8-DOβ-K225R-expressing cells. (C) Lysates prepared from transfected cells above were immunoprecipitated with nonsaturating amounts of anti-CD8 antibody OKT8. After PAGE electrophoresis and membrane transfer blots were probed with anti-ubiquitin-HRP antibody. The position of ubiquitinated CD8-DO is indicated. Ubiquitinated MARCH proteins running above 70 kDa have been previously characterised 26. (D) As in (C) but probed with anti-CD8 as a loading control. Although less CD8 was present in lane 8, repeat experiments confirm the lack of ubiquitinated products associated with CD8-DOβ-K225R. (E) As in (C) but probed with anti-EGFP antibodies to detect co-precipitated MARCH proteins. The position of MARCH1-GFP and MARCH8-GFP is indicated (arrow heads). The difference in migration corresponds to the difference in molecular weight between MARCH1 and MARCH8, as previously characterised 26. Data shown are representative of three independent experiments performed.

To investigate ubiquitination of HLA-DO, we generated a chimeric CD8 reporter construct comprising the extracellular domains of CD8 and the transmembrane and cytoplasmic tail of DOβ. This was detected at the cell surface when transfected into HEK 293T cells (Fig. 1A). In cells co-transfected with MARCH1, MARCH8, or MARCH9, levels of surface CD8-DOβ were greatly reduced. Expression of a catalytically inactive MARCH8 construct did not influence surface expression (Fig. 1A). The cytoplasmic tail of HLA-DOβ contains a single lysine residue at position 225 of the mature protein. This residue is well conserved in mammalian species (Supporting Information Fig. 1). Substitution of this residue for arginine (CD8-DOβ-K^225^R), a conservative change for another positively charged amino acid, largely prevented MARCH9-induced downregulation (Fig. 1B). Some influence of MARCH1 and MARCH8 was still observed but downregulation was greatly reduced.

### MARCH-induced internalisation of CD8-DOβ correlates with polyubiquitination of residue K225

**Figure 2:**
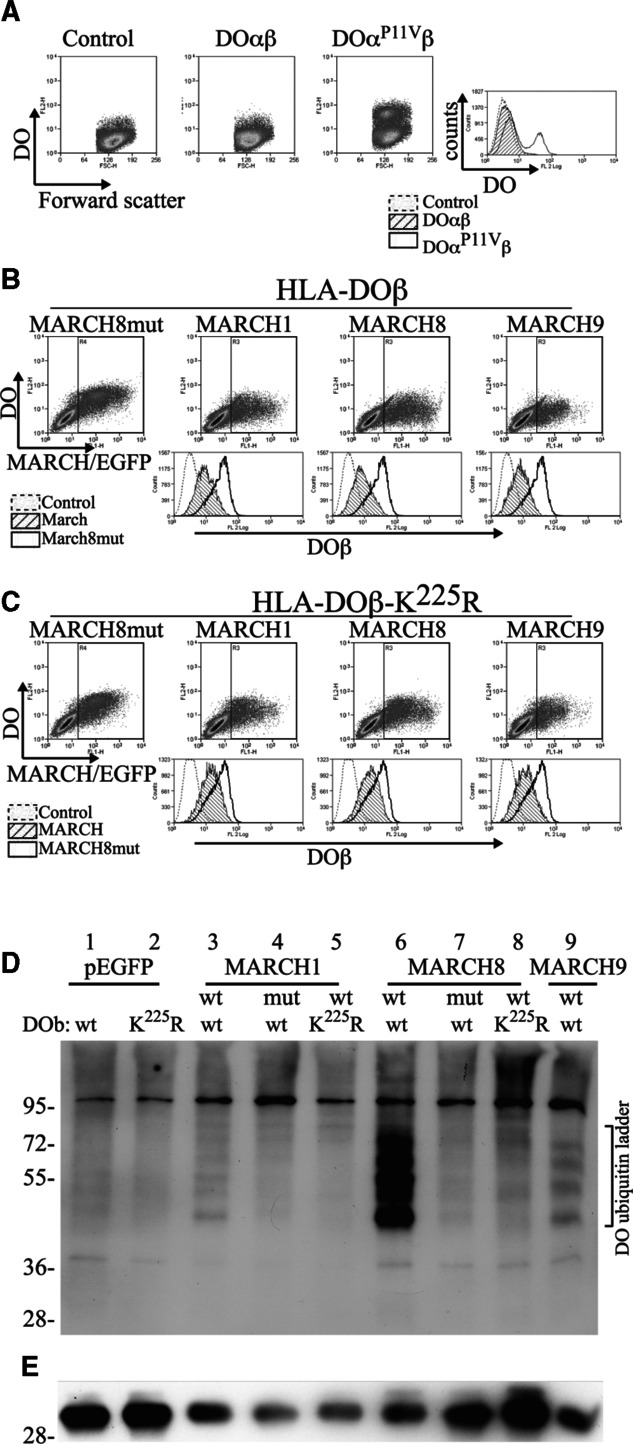
Surface expression and ubiquitination of DOα^P11V^/β in the presence of MARCH family E3 ligases. (A) HEK 293T cells were transfected with wild-type DOαβ or DOα^P11V^/β and surface expression of DO determined by flow cytometry with anti-DO antibody Mags.DO5. (B) HEK 293T cells were transfected with DOα^P11V^/β together with active and inactive MARCH-EGFP E3 ligase constructs. Cells were prepared for analysis and surface levels of DO determined with Mags.DO5. Dot plots and histogram profiles show wild-type CD8-DOβ surface expression in the presence of MARCH1, MARCH8, MARCH9 and catalytically inactive MARCH8mut. (C) As above but with DOα^P11V^/β-K225R expressing cells. (D) Lysates were prepared from transfected cells above and immunoprecipitated with nonsaturating amounts of anti-DO antibody Mags.DO5. After PAGE electrophoresis and membrane transfer blots were probed with anti-ubiquitin-HRP antibody. (E) As above but probed with anti-DOβ antiserum as a loading control. The data shown are representative of three independent experiments performed.

Immunoprecipitation and western blotting was used to determine if the reporter molecule was directly ubiquitinated by the MARCH E3 ligases (Fig. 1C). Complex patterns of polyubiquitination were seen in immunoprecipitates from cells cotransfected with MARCH1 and MARCH8 (Fig. 1C, lanes 3 and 6). Substitution of K225 for arginine (CD8-DOβ-K^225^R) resulted in loss of the ubiquitin ladder in the 36–72 kDa molecular weight range (Fig. 1C, lanes 5 and 8). Bands of this size correspond to the ubiquitinated CD8-DOβ reporter molecule. MARCH proteins that co-immunoprecipitate with DO were detected by Western blotting with anti-GFP antibody (Fig. 1E) and anti-ubiquitin antibody (Fig. 1C). Ubiquitinated MARCH protein appears as higher molecular weight products in the 72–95 kDa size range (Fig. 1C). This was more evident with MARCH1 than MARCH8. Expression of a catalytically inactive MARCH8 construct (MARCH8-mut) did not facilitate ubiquitination of CD8-DOβ (Fig. 1C, lane 7). MARCH8-mut has the zinc finger-forming cysteine residues (C80, 83, 123 and 125) mutated to serine and this destroys the catalytic activity of the RING-CH domain. Interestingly, an analogous MARCH1 mutant, in which the zinc finger-forming cysteine residues (C63, 66, 106 and 109) were mutated to serine still enabled ubiquitination of CD8-DOβ, although at reduced levels (Fig. 2D, lane 4). Additional MARCH1 constructs with alternative mutations in the catalytic domain were also partially active (data not shown), suggesting that substrate ubiquitination still occurred when the RING-CH domain was inactivated. The reason for this residual activity is not known but could relate to the capacity of MARCH1 to dimerise with other ligases 21. It is notable that the MARCH1-mut that co-precipitates with CD8-DOβ is itself heavily ubiquitinated demonstrating that ligase activity is recruited to the DO-MARCH protein complex (Fig. 1C, lane 4).

### MARCH1, 8 and 9 influence localisation of HLA-DO through direct and indirect mechanisms

HLA-DO requires HLA-DM for correct folding and egress from the ER 3. To analyse ubiquitination of HLA-DO independently of its association with HLA-DM, we generated a mutant DOα construct bearing a proline to valine substitution. This allows DM-independent egress of DO from the ER 6. We confirmed that DOα^P11V^/β dimers were well expressed at the cell surface of transiently transfected cells compared to cells expressing wild-type DOα/β (Fig. 2A). This allowed us to use alteration of surface DO expression as a proxy for ubiquitination. Co-expression of DOα^P11V^/β with MARCH1, MARCH8, or MARCH9 resulted in downregulation of surface expressed DO (Fig. 2B). Wild-type MARCH1, MARCH8 and MARCH9 were all able to induce ubiquitination of DOα/β dimers, although MARCH8 was by far the most efficient (Fig. 2D). The loading control shows that similar amounts of DOα^P11V^/β are present in tracks 3, 6 and 9 and allows comparison of the efficiency of ubiquitination by MARCH1, MARCH8 and MARCH9. By western blot, we were unable to detect MARCH proteins in immunoprecipitates of DOα^P11V^/β. This is likely due to the lower level of DO expression compared with that of CD8-DOβ, where the association is clear. Unexpectedly, significant downregulation was still observed in constructs lacking lysine 225 (DOα/DOβ-K^225^R (Fig. 2C), even though ubiquitination by MARCH1 and MARCH8 was negligible (Fig. 2D, lane 4 and 7). This suggested that indirect mechanisms could also contribute to regulation of DO by MARCH proteins. We attempted to directly demonstrate ubiquitination of DO in immunoprecipitates from Raji cells and peripheral blood B cells without success. This is most likely due to the low level expression of DO, which is expressed at 1/20th the level of DR 15, and the poor reactivity of antibody reagents for DO compared with those for DR. We are continuing to investigate this using Ig-crosslinked and PMA-activated B cells but have been unsuccessful to date 22. The most obvious situation where posttranslational regulation of DO levels occurs, independently of DM, is in GC centroblasts and centrocytes. Alternative strategies will have to be developed to investigate this due to the technical limitations involved in working with GC cells.

### Di-leucine and tyrosine-based internalisation motifs influence MARCH-induced trafficking of HLA-DO

**Figure 3:**
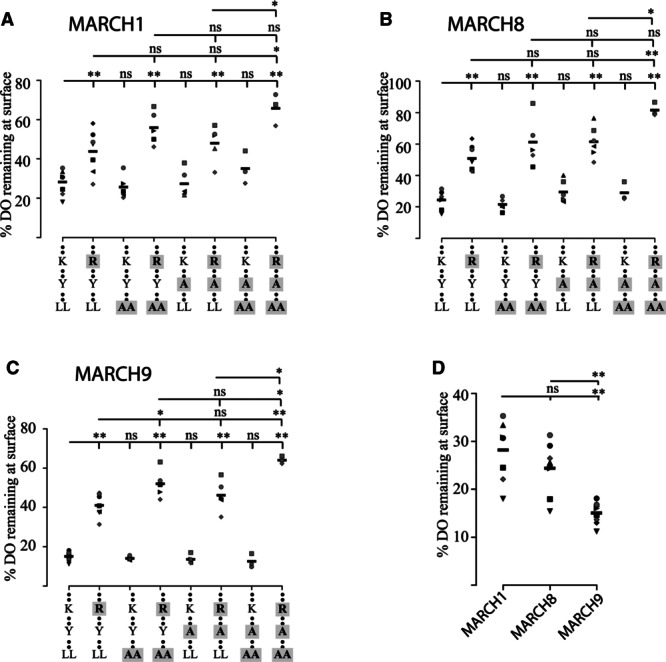
Lysine, tyrosine and di-leucine motifs all contribute to MARCH-induced downregulation of HLA-DO. HEK 293T cells were transfected with wild-type DOα^P11V^/β and constructs bearing K225R, Y227A and LL242, 243AA substitutions in the DOβ cytoplasmic tail. Surface DO was measured by flow cytometry using anti-DO antibody Mags. DO5. The percent surface expression of DO constructs in the presence of (A) MARCH1, (B) MARCH8 and (C) MARCH9 are shown. Wild-type DOα^P11V^/β is shown in lane 1. Substitutions to the cytoplasmic tail of DOβ are shown in bold and highlighted. Each construct was analysed in at least three replicate experiments. Different replicate experiments are denoted by different symbols. (D) Comparison or the relative downregulation of DOα^P11V^/β by MARCH1, MARCH8 and MARCH9. Surface expression (%) = (MFI cells expressing E3 ligase × 100)/(MFI cells transfected with GFP vector alone). *p < 0.05, **p < 0.01, ns, not significant, *t*-test. Single amino acid code is used.

The DOα cytoplasmic tail is short and contains no known endocytosis motifs 1. In comparison, the cytoplasmic tail of DOβ includes di-leucine and tyrosine-based endocytosis motifs that function independently and in combination 15, 23. To investigate if these elements were involved in MARCH-dependent DO downregulation, they were mutated in combination with the K^225^R substitution. In the absence of K225, simultaneous mutation of both the tyrosine and di-leucine motifs resulted in significantly greater expression of DO at the cell surface (Fig. 3 A–C, column 8). This was statistically significant for all three MARCH proteins. Individual mutation of the dileucine and tyrosine motifs also resulted in greater expression at the cell surface but this was not statistically significant, except in the case of the dileucine motif and MARCH9 (Fig. 3C, column 4). Thus, MARCH-induced DO downregulation was due, at least in part, to indirect effects that involved endocytic-targeting motifs.

When lysine 225 was available for ubiquitination, the Y^227^A and LL^242,243^AA substitutions had no statistically significant effect on MARCH-induced downregulation (Fig. 3A–C, compare columns 1, 3, 5 and 7). A likely explanation for this is that ubiquitination of K225 is dominant and with MARCH overexpression, more subtle influences afforded by the tyrosine and di-leucine motifs are masked.

Comparison of results with constructs lacking both K225 and Y227 with the construct mutated for all three motifs (K225, Y227 and LL242,243) demonstrated that the di-leucine motif had a significant impact on MARCH-induced downregulation (Fig. 3A–C, compare columns 6 and 8). Whilst comparison of the construct lacking K225 and LL242,243 with that mutated for all three motifs (K225, Y227 and LL242,243) showed no significant impact on MARCH1 or MARCH8-induced downregulation (Fig. 3A and B, compare columns 6 and 8). This suggests an order of standing with K225 being the single most important motif followed by the di-leucine and finally the tyrosine motif. Importantly, together the data show that MARCH1, MARCH8 and MARCH9 can influence trafficking of HLA-DO in the absence of direct ubiquitination, probably through indirect effects on components of the endocytic machinery that regulate trafficking of DO through di-leucine and tyrosine-based motifs.

We next determined if all three MARCH proteins targeted DO with the same efficiency. As shown in Figure 3D, MARCH9 was significantly more efficient compared to MARCH1 or 8. Thus, although MARCH8 was associated with the highest level of DO-directed ubiquitination, MARCH9 was more efficient at relocating DO from the cell surface. Interestingly, MARCH9 is restricted in its recognition of MHCII and specifically targets DQ whilst having little effect on DR or DP 24.

### Ubiquitination by the various MARCH proteins has different consequences for HLA-DO

**Figure 4:**
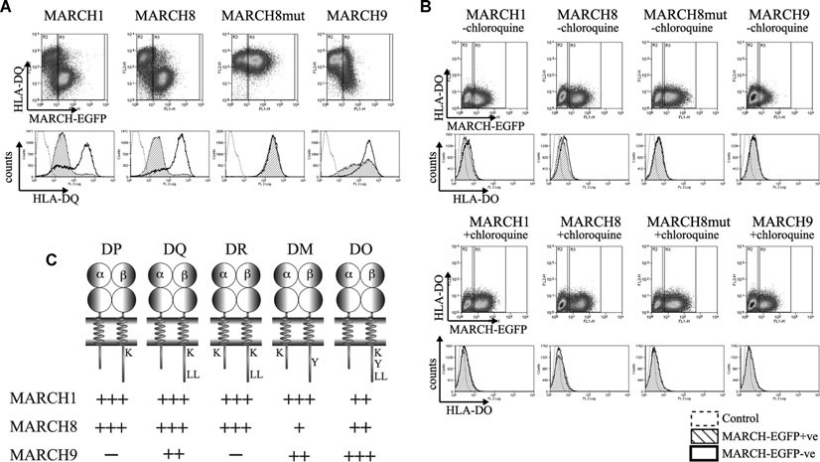
MARCH1 and MARCH8 induce degradation of HLA-DO. (A) Raji cells were transduced with MARCH1, MARCH8, MARCH9 and MARCH8-mut and surface HLA-DQ monitored by flow cytometry using anti-DQ antibody L2DQ to confirm activity of the transfected E3 ligases. (B) Intracellular staining for DO performed on saponin-permeabilised Raji cells using anti-DO antibody Mags. DO5. Levels of DO were assessed by flow cytometry. Reduced intracellular staining is seen in cells transduced with MARCH1 and MARCH8 in the absence of chloroquine. In the presence of chloroquine levels of intracellular DO remain unaltered. Data shown are representative of at least three independent experiments performed. (C) Schematic diagram summarising ubiquitination sites and E3 ligases involved in regulating components of the class II antigen presentation machinery. MARCH1 and MARCH8 regulate surface expression of DP, DQ and DR 20, 35. MARCH9 specifically targets DQ 24, 33 and DRα can also be ubiquitinated 25. HLA-DM is regulated directly by ubiquitination of a DMα encoded lysine residue and indirectly through a mechanism involving the tyrosine-based sorting motif 26. DO is regulated by ubiquitination of the β chain-encoded lysine residue and indirectly through the di-leucine and tyrosine motifs.

We investigated the consequence of ubiquitination of DO by the different MARCH proteins. Raji cells expressing endogenous DO were transduced with MARCH1, MARCH8 and MARCH9 and intracellular DO levels measured by flow cytometry. Cells expressing the various MARCH proteins showed reduced HLA-DQ surface expression confirming the efficiency of transduction (Fig. 4A). HLA-DQ was monitored as all three MARCH proteins influence surface expression of this MHCII isotype. Cells transduced with MARCH1 and MARCH8 showed reduced intracellular DO staining (Fig. 4B) suggesting degradation of DO. No reduction was seen in cells transduced with MARCH9 or MARCH8-mut. In all cases, levels of DO remained constant in the presence of chloroquine, an inhibitor of lysosomal degradation (Fig. 4B). Over three independent experiments levels of DO in MARCH8 transfected cells were 30.75% lower in the absence compared to presence of chloroquine. For MARCH1 and MARCH9, the comparable figures were 17.9% and 0.95%, respectively. Therefore, MARCH1 and MARCH8 induce lysosomal degradation of HLA-DO, whereas MARCH9, although having the greatest influence on internalisation of DO does not target molecules for degradation. We also noted that MARCH9 itself appeared to be targeted for lysosomal degradation as levels of MARCH9-EGFP increased significantly upon chloroquine treatment (Fig. 4B). This was less obvious for the other MARCH proteins.

From these and previous studies, it is apparent that poly-ubiquitination is a common posttranslational modification of both classical and nonclassical MHC molecules 18–20, 24–26. In Figure 4C, we summarise schematically what is known regarding ubiquitination sites in components of the class II antigen presentation machinery. We show that all classical and nonclassical MHCII molecules are subject to polyubiquitination by a range of MARCH E3 ligases. These may function through direct ubiquination of the MHCII molecule itself or indirectly through mechanisms that probably involve ubiquitination of components of the endocytic machinery.

## Discussion

The cytoplasmic tail of HLA-DOβ encodes a single lysine residue that is highly conserved across species, implying an important functional role. It is also present in an identical location in classical MHCII molecules HLA-DP, DQ and DR, which again suggests an important, possibly shared function. For MHCII, at least one defined role is clear. Ubiquitination of this residue by MARCH family E3 ligases regulates surface expression of pMHCII in maturing professional APCs 18–20. Here we investigated ubiquitination of DO and determined that although it was co-regulated by the same MARCH E3 ligases that target all MHCII, it also had the capacity for independent regulation by MARCH9.

We confirmed that the conserved lysine in DO was poly-ubiquitinated by MARCH1 and MARCH8, two related E3 ligases that target classical MHCII. This was associated with reduced surface DO expression and targeting to lysosomal compartments for degradation, very similar to what is known to occur with DR. However, DO was also subject to more specific regulation by MARCH9, a more distantly related MARCH homologue. Targeting of DO by MARCH9 was significantly more efficient than with MARCH1 or MARCH8. Interestingly MARCH9 shows very little capacity to regulate DR or DP, although it does interact with DQ 24. In Figure 4C, we provide a schematic summary of MHCII ubiquitination. This shows the location of ubiquitinated residues present in the various molecules and indicates the efficiency with which different E3 ligases cause redistribution from the cell surface. In essence, MARCH1 and MARCH8 efficiently target MHCII and to a lesser extent DM and DO. MARCH9 in contrast shows greatest specificity for DO but also targets DM and DQ albeit less efficiently.

Why would DO require MARCH regulation and how might this occur? Several possibilities are worthy of discussion and these are centered on the major roles of ubiquitination; namely protein degradation and trafficking. First, expression of DO is highly specific and under particularly complex control in developing B cells. It is here that MHCII, DM and DO expression is most dynamic and it is also where DO is proposed to exert its main biological activity. Briefly, MHCII and DM are expressed through pro-B, pre-B and into immature and mature B-cell stages. DO expression in comparison is initiated as cells mature into IgD^+^/CD40^+^ B cells and then during germinal center reactions expression is reduced. DO levels continue to fall as the cells develop into centroblasts and centrocytes, whereas DM expression remains relatively constant 7, 27. This pattern is unique to GC reactions because in general downregulation of DO is always associated with reduced DM expression, as is the case for Ig-crosslinking or PMA activation 22. Importantly, the downregulation of DO in GCs is by posttranslational mechanisms, as mRNA transcripts remain unchanged 27. The ability of MARCH9 to specifically target DO would provide a mechanism to achieve this. However in our hands, although the major target of MARCH9 was DO, ubiquitination did not appear to lead to lysosomal degradation (Fig. 4B). Further studies using GC-derived centroblasts and centrocytes cells will be required to see if MARCH9 is involved in DO downregulation in these cells.

A second possible role for DO ubiquitination relates to localisation. Ubiquitination has well-recognised roles in endocytosis and sorting (reviewed in 28) and may therefore play a role in localisation of DO/DM and MHCII in endosomal compartments. In general, receptor ubiquitylation promotes targeting into the luminal vesicles of MVBs that facilitates protein degradation 28. DO has been shown to enhance the incorporation of DO/DM complexes and MHCII into the perimeter membrane of MVBs fostering interaction of these molecules without their degradation. This capability requires the cytoplasmic tail of DOβ 15. MARCH9-induced ubiquitination of DO did not result in degradation but merely enhanced intracellular localisation. This would be expected if it was directed to the perimeter membrane of MVBs rather than to luminal vesicles. DO in complex with DM has been shown to be recycled back to the MIICs from the plasma membrane 15, 29. Ubiquitination by MARCH9 could facilitate this without the degradation that is associated with regulation by MARCH1 or MARCH8.

Although we confirmed that the lysine residue was the sole target for ubiquitination in DO, when this residue was substituted for arginine, significant MARCH-induced downregulation of DO was still observed. This suggested that the MARCH proteins were still influencing trafficking of DO in the absence of direct ubiquitination. We determined that this effect required the presence of tyrosine and di-leucine-based endocytosis motifs located in the tail of DOβ. Combinatorial mutation of the lysine, tyrosine and di-leucine motifs showed that the lysine was the single most important factor. In the absence of this residue, mutation of the di-leucine or tyrosine motifs had no significant influence on MARCH1 or MARCH8-induced downregulation. However, simultaneous mutation of both tyrosine and di-leucine motifs significantly impaired downregulation in all cases. Ubiquitination of epsin, Eps15 and ESCRT sorting components that regulate endocytosis has been described (reviewed in 30). We propose that the MARCH proteins influence ubiquitination of sorting adaptors and hence modulate internalisation of HLA-DO indirectly by influencing the activity of the endocytic machinery. A similar “ubiquitin-dependent endocytosis motif” directs internalisation of the human growth hormone receptor in the absence of direct receptor ubiquitination 31.

After considerable study, the biological role of DO still remains uncertain. However, the highly dynamic expression of DO in GC B-cells points to a function in antibody maturation. Posttranslational DO regulation occurs during this process and data presented here suggest that MARCH family E3 ligases are strong candidates to undertake this role. The fact that all components of the MHCII presentation machinery, MHCII 19, DM 26 and now DO (summarised in Fig. 4) can be modified by ubiquitination demonstrates that this is an important mechanism regulating complex aspects of MHC class II peptide presentation.

## Materials and methods

### Antibodies

The following antibodies were used in this study; anti HLA-DQ antibody L2DQ (Cancer Research Technology), anti-ubiquitin-HRP P4D1 and rabbit anti-CD8α polyclonal H-160 (Santa Cruz Biotechnology), anti-CD8α OKT8 (ATCC), anti-DOβ DOB.L1 32 anti-DO Mags.DO5 27, anti-EGFP JL-8 (Clontech), anti-mouse TrueBlot-HRP eB144/7A7 (eBioscience), rabbit anti-sheep-HRP (Dako), and rabbit anti-mouse RPE (Thermo Scientific). Sheep anti-DOβ antiserum generated against recombinant full-length human DOβ.

### Plasmid constructs

The CD8-DOβ reporter construct comprised residues 1–176 of the luminal domain of CD8α and the transmembrane and cytoplasmic tail of DOβ (residues 194–247). This was generated by overlap PCR using KOD HiFi polymerase (Calbiochem). Alterations to the sequence were introduced using overlapping primer pairs. Constructs were ligated into pcDNA3.1/Zeo^+^ or/Neo^−^ (Invitrogen) and authenticated by DNA sequencing. The amino acid composition of relevant regions of constructs used in this study are summarized in Table 1. Plasmids pEGFPc1-MARCH1, pEGFPc1-MARCH9, pCMV8.91, pMD-G and pHR'SIN-cPPT-SGW were provided by Paul Lehner (Cambridge, UK). Plasmids pEGFPc2-MARCH1mut, pEGFPc2-MARCH8wt and MARCH8mut have previously been described 26. DOα and DOβ constructs were amplified by PCR and cloned into pCDNA3.1 Neo^−^.

**Table 1:** Composition of DO constructs used in this study

Construct	Cytoplasmic taila)
CD8-DOβ	^222^RAQKGYVRTQMSGNEVSRAVLLPQSC^247^b), c)
CD8-DOβ-K^225^R	^222^RAQ**R**GYVRTQMSGNEVSRAVLLPQSC^247^
DOα	^216^MGTYVSSVPR^225^
DOα^P11V^	^216^MGTYVSSVPR^225^
DOβ	^222^RAQKGYVRTQMSGNEVSRAVLLPQSC^247^
DOβ-K^225^R	^222^RAQ**R**GYVRTQMSGNEVSRAVLLPQSC^247^
DOβ-LL^242,243^AA	^222^RAQKGYVRTQMSGNEVSRAV**AA**PQSC^247^
DOβ-K^225^R,LL^242,243^AA	^222^RAQ**R**GYVRTQMSGNEVSRAV**AA**PQSC^247^
DOβ-Y^227^A	^222^RAQKG**A**VRTQMSGNEVSRAVLLPQSC^247^
DOβ-K^225^R,Y^227^A	^222^RAQ**R**G**A**VRTQMSGNEVSRAVLLPQSC^247^
DOβ-Y^227^A,LL^242,243^AA	^222^RAQKG**A**VRTQMSGNEVSRAV**AA**PQSC^247^
DOβ-K^225^R,Y^227^A,LL^242,243^AA	^222^RAQ**R**G**A**VRTQMSGNEVSRAV**AA**PQSC^247^

a) Substituted residues in the cytoplasmic tail of DOβ are in bold and highlighted.

b) Numbering is taken from the mature protein and does not include the signal sequence.

c) Single amino acid code is used.

### Flow cytometry

FACS analysis was as previously described 33. Constructs were transfected into HEK 293T cells and cells harvested and analysed 24–36 h later 26, 34. Due to residual activity associated with MARCH1-mut and MARCH9-mut constructs inactive MARCH8-mut was used as the expression control. This was validated by comparison of surface CD8-reporter expression alone or in the presence of cotransfected MARCH8-mut or GFP. Neither MARCH8-mut or GFP significantly influenced CD8-reporter surface expression. Raji cells were grown in RPMI 1640 supplemented with 10% FCS and 2 mM alanine-glutamine dipeptide. After transduction (36 h), chloroquine (to 60 uM) was added and cells were grown for an additional 12 h. For intracellular staining, cells were fixed in 3% formaldehyde/PBS for 15 min, washed and then permeabilised with 0.2% saponin in FACS buffer. Fc receptors were blocked with 40% human AB serum (Sigma) for 10 min and stained as above but in the presence of 0.2% saponin. Acquisition of data was performed using an FACScan flow cytometer (BD Biosciences) and data analysed with Summit v4.3 software. The expression of surface protein levels was calculated using mean fluorescent intensity (MFI) in the presence of catalytically active E3 ligase as monitored through expression of GFP. Surface expression (%) = (MFI of cells expressing E3 ligase X 100)/(MFI of cells transfected with march8mut or untransfected cells). Statistical analysis was performed using a two-tailed, unpaired Student's *t*-test. Differences were classed as not significant (NS), significant (*p<0.05) or highly significant (^**^p<0.01).

### Immunoprecipitation and western blotting

Immunoprecipitation and western blotting were as previously described 33. Protein was precipitated with appropriate antibodies and Protein A Agarose (Sigma) using standard procedures. To ensure similar amounts of CD8-DO and DO were immunoprecipitated from transient transfections nonsaturating amounts of antibody were used. Immunoprecipitates were subject to SDS-PAGE analysis and Western transfer onto PVDF membrane (Millipore). Ubiquitination was detected using anti-ubiquitin-HRP and rapid immunodetection reagents (ECL+) according to manufacturer's instructions (GE Healthcare).

## Abbreviations

GC: germinal center
Ii: invariant chain
MARCH: membrane-associated RING-CH
MFI: mean fluorescent intensity
MVB: multivesicular body
pMHCII: peptide-loaded MHC class II

## Supplementary material

As a service to our authors and readers, this journal provides supporting information supplied by the authors. Such materials are peer reviewed and may be re-organized for online delivery, but are not copy-edited or typeset. Technical support issues arising from supporting information (other than missing files) should be addressed to the authors.
