# Supplementary material for: Ubiquitination of HLA-DO by MARCH family E3 ligases
Source: Eur J Immunol. 2013 Feb 11;43(5):1153–61. doi: 10.1002/eji.201243043 (PMC3655539; doi:10.1002/eji.201243043)
Supplement: Supplementary file 2 [file eji0043-1153-SD2.pdf]

European Journal of Immunology

Supporting Information

for

DOI 10.1002/eji.201243043

Martin Jahnke, John Trowsdale and Adrian P. Kelly

Ubiquitination of HLA-DO by MARCH family E3 ligases

© 2013 WILEY-VCH Verlag GmbH & Co. KGaA, Weinheim

Supplemental Figure 1. **Lysine residues are conserved in the cytoplasmic tail of mammalian DOβ molecules.**
Amino acid sequences of the cytoplasmic tails of the homologues of HLA-DOα and β from 24 mammals are shown. The ubiquitination site at the residue corresponding to human DOβ lysine 225 is conserved across most mammalian species. The C-terminal parts of the putative transmembrane domains are highlighted in grey, lysine residues are shown in red, and YxxΦ and dileucine motifs are in bold print. Sequences were obtained from the Ensemble database, Release 62. All species for which both α and β chains were available are shown. In orangutan, mouse lemur, horse, dog and elephant, the DOβ STOP codon was not found in the database (…).

**Species**	**Species name**	**DOα**	**DOβ**
Human	*Homo sapiens*	…LVGTVLIIMGTYVSSVPR	…LVGIVIQLRAQKG**YVRT**QMSGNEVSRAV**LL**PQSC
Chimanzee	*Pan troglodytes*	…LVGTVLIIVGTYVSSVPR	…LVGIVIQLRAQKG**YVRT**QMSGNEVSRAV**LL**PQSC
Orang Utan	*Pongo pygmaeus*	…LVGTVLIIMGTYVSSARR	…LVGIVIQLRAQKG**YVRT**QMSGNEVSRAV**LL**PQ[…]
Macaque	*Macaca mulatta*	…LVGTILIIMGTYVSSAPR	…LMGIIIQLRAQKG**YVRT**QMSGDEVSRAV**LL**PQPC
Marmoset	*Callithrix jacchus*	…LVGTILIIMGICVSSAPR	…LVGIVIQLRAQKGHVGTHMSGDEVSRAV**LL**PQSY
Galago	*Otolemur garnettii*	…LVGTILIIKGTCQSSAPR	…LVGIVIHLRAQKG**YMEI**QKSGDEVSRAV**LF**PQPH
Mouse Lemur	*Microcebus murinus*	…LVGTALVITGTRLSSAPGQ	…LLLVGVLLRRRAQR[…]
Tarsier	*Tarsius syrichta*	…LVGTILIIRGTCESHAPR	…LVGIVIHLRARKG**YMDT**QQSGDEVPRTVMPWQPC
Large Flying Fox	*Pteropus vampyrus*	…LVGTVLIITSTCLHSAPR	…LVGIAVHLRAQKG**YAET**QLSGDKVSRSVLPPLTY
Pika	*Ochotona princeps*	…LQGTVLILIATRMSRAPRQ	…LVGIITYLRAWKGCMETQRSAAEVSRAGFPSQPY
Mouse	*Mus musculus*	…LLGTVLMITGTRRPSIRR	…LVGVVIHLKAQKASVETQ-PGNEASRESLHSQP.
Rat	*Rattus norvegicus*	…LLGTLLMVTATRMPSTRR	…LVGAVIHLKAQKASVETQ-PGNEASRA**LL**PPHPY
Kangaroo Rat	*Dipodomys ordii*	…LMGTILIILATTMADAHR	…LAGLIIHLRAWKGSAEAQ-AGHEVSRA**LL**PSQPY
Ground Squirrel	*Spermophilus tridecemlineatus*	…LMGTILIITGTCMSSCPR	…LVGIIIHLRARKGSVETQ-SGDKISRA**IL**QKPNS
Horse	*Equus caballus*	…LVGTILLIISTCLSGAPR	…LVGIFIHLRARKG[…]
Pig	*Sus scrofa*	…LVGVTLIITGRRRSSSPR	…LVGIIIHVRARKGRVETQLSGDEPH
Alpaca	*Vicugna pacos*	…LVGIVLIITGSRLSRAPR	…LVGITVHVRARKG**YVET**QLSGDKVLGAVCPEQPH
Bottlenose Dolphin	*Tursiops truncatus*	…LVGVILIITGTCLSSTPR	…LVGIVIHTRARKM--------VTVPRAVLRPQPH
Dog	*Canis lupus*	…LVGTIFIIRGTCLSSGPRYRGPQ	…LVGTVICLRAQKG**YVET**QFSGDEVM[…]
Cat	*Felis catus*	…LVGFLVGTILIIRGTCLSSAPR	…LVGTVICLRAQKG**YMET**QLSGDEVSRAV-PSPPH
Nine-Banded Armadillo	*Dasypus novemcinctus*	…LAGLVVGTILIFAGTRLSSAPR	…LVGIIICLRARKG**YMET**QLSNDGVSRA**LL**PPQLYQ
African Elephant	*Loxodonta africana*	…LVGTIFIISGTCLSSTPRCRGPR	…LVGIVIHFRGQKG[…]
Hyrax	*Procavia capensis*	…LVGTIFIISGACLSSDPK	…LVGIFIYFRAQKG**YVKT**QLSVDKVSRSVLPPQPYE
Lesser Hedgehog Tenrec	*Echinops telfairi*	…LLGTVLIIHSTCCSSVLR	…LVGICIRLKAQRGHVETPQLHDEGSRSV-PIQSC
